# Supplementary material for: Modeling glioblastoma heterogeneity as a dynamic network of cell states
Source: Mol Syst Biol. 2021 Sep 16;17(9):e10105. doi: 10.15252/msb.202010105 (PMC8444284; doi:10.15252/msb.202010105)
Supplement: Supplementary file 6 — Source Data for Figure 5 [file MSB-17-e10105-s004.zip › Figure5A_sourcedata/GSEA_3017/hallmarks_stateF.GseaPreranked.1621934628066/HALLMARK_MYC_TARGETS_V2.html]

Details for gene set HALLMARK\_MYC\_TARGETS\_V2[GSEA]

|  || Dataset | state33017 |
| Phenotype | NoPhenotypeAvailable |
| Upregulated in class | na\_neg |
| GeneSet | HALLMARK\_MYC\_TARGETS\_V2 |
| Enrichment Score (ES) | -0.6887003 |
| Normalized Enrichment Score (NES) | -2.599234 |
| Nominal p-value | 0.0 |
| FDR q-value | 0.0 |
| FWER p-Value | 0.0 |
Table: GSEA Results Summary

  

Fig 1: Enrichment plot: HALLMARK\_MYC\_TARGETS\_V2      
 Profile of the Running ES Score & Positions of GeneSet Members on the Rank Ordered List

  

| PROBE | GENE SYMBOL | GENE\_TITLE | RANK IN GENE LIST | RANK METRIC SCORE | RUNNING ES | CORE ENRICHMENT || 1 | NOLC1 |  |  | 1542 | -0.270 | -0.6452 | Yes |
| 2 | NPM1 |  |  | 1543 | -0.270 | -0.6017 | Yes |
| 3 | IMP4 |  |  | 1553 | -0.273 | -0.5618 | Yes |
| 4 | TMEM97 |  |  | 1629 | -0.293 | -0.5481 | Yes |
| 5 | GNL3 |  |  | 1716 | -0.324 | -0.5343 | Yes |
| 6 | PLK4 |  |  | 1740 | -0.336 | -0.4906 | Yes |
| 7 | HSPD1 |  |  | 1742 | -0.336 | -0.4368 | Yes |
| 8 | DDX18 |  |  | 1785 | -0.355 | -0.3984 | Yes |
| 9 | SRM |  |  | 1786 | -0.355 | -0.3411 | Yes |
| 10 | WDR43 |  |  | 1920 | -0.438 | -0.3300 | Yes |
| 11 | NOP56 |  |  | 1953 | -0.456 | -0.2707 | Yes |
| 12 | MCM4 |  |  | 1993 | -0.487 | -0.2097 | Yes |
| 13 | PLK1 |  |  | 2087 | -0.624 | -0.1508 | Yes |
| 14 | MRTO4 |  |  | 2113 | -0.683 | -0.0519 | Yes |
| 15 | PA2G4 |  |  | 2123 | -0.707 | 0.0581 | Yes |
Table: GSEA details [plain text format]

  

Fig 2: HALLMARK\_MYC\_TARGETS\_V2: Random ES distribution      
 Gene set null distribution of ES for **HALLMARK\_MYC\_TARGETS\_V2**
